# Microdeletions in 9q33.3-q34.11 in five patients with intellectual disability, microcephaly, and seizures of incomplete penetrance: is *STXBP1* not the only causative gene?

**DOI:** 10.1186/s13039-015-0178-8

**Published:** 2015-09-29

**Authors:** Julia K. Ehret, Hartmut Engels, Kirsten Cremer, Jessica Becker, Johannes P. Zimmermann, Eva Wohlleber, Ute Grasshoff, Eva Rossier, Michael Bonin, Elisabeth Mangold, Andrea Bevot, Stefanie Schön, Stefanie Heilmann-Heimbach, Nicola Dennert, Michèle Mathieu-Dramard, Elodie Lacaze, Ghislaine Plessis, Alain de Broca, Guillaume Jedraszak, Benno Röthlisberger, Peter Miny, Isabel Filges, Andreas Dufke, Joris Andrieux, Jennifer A. Lee, Alexander M. Zink

**Affiliations:** Institute of Human Genetics, University of Bonn, Sigmund-Freud-Strasse 25, 53105 Bonn, Germany; Department of Genomics, Life & Brain Center, University of Bonn, Bonn, Germany; Present Address: Center for Human Genetics, Freiburg, Germany; Institute of Medical Genetics and Applied Genomics, University of Tübingen, Tübingen, Germany; Present Address: Genetikum, Neu-Ulm, Germany; Present Address: IMGM Laboratories GmbH, Martinsried, Germany; Children’s Hospital, University of Tübingen, Tübingen, Germany; MVZ Dr. Eberhard & Partner, Dortmund, Germany; Centre de Génétique, CHU d’Amiens, Amiens, France; Service de génétique, CHU de Caen, Caen, France; Service de Neurologie Pédiatrique, CHU d’Amiens, Amiens, France; Medical Genetics, Centre of Laboratory Medicine, Cantonal Hospital Aarau, Aarau, Switzerland; Medical Genetics, University Hospital Basel, Basel, Switzerland; Laboratoire de Génétique Médicale, Hôpital Jeanne de Flandre, CHRU de Lille, Lille, France; Present Address: Greenwood Genetic Center, Greenwood, SC USA

**Keywords:** Microdeletion 9q33.3-q34.11, Intellectual disability, *STXBP1*, Contiguous gene syndrome, Haploinsufficiency

## Abstract

**Background:**

Most microdeletions involving chromosome sub-bands 9q33.3-9q34.11 to this point have been detected by analyses focused on *STXBP1*, a gene known to cause early infantile epileptic encephalopathy 4 and other seizure phenotypes. Loss-of-function mutations of *STXBP1* have also been identified in some patients with intellectual disability without epilepsy*.* Consequently, *STXBP1* is widely assumed to be the gene causing both seizures and intellectual disability in patients with 9q33.3-q34.11 microdeletions.

**Results:**

We report five patients with overlapping microdeletions of chromosome 9q33.3-q34.11, four of them previously unreported. Their common clinical features include intellectual disability, psychomotor developmental delay with delayed or absent speech, muscular hypotonia, and strabismus. Microcephaly and short stature are each present in four of the patients. Two of the patients had seizures. *De novo* deletions range from 1.23 to 4.13 Mb, whereas the smallest deletion of 432 kb in patient 3 was inherited from her mother who is reported to have mild intellectual disability. The smallest region of overlap (SRO) of these deletions in 9q33.3 does not encompass *STXBP1*, but includes two genes that have not been previously associated with disease, *RALGPS1* and *GARNL3.*

Sequencing of the two SRO genes *RALGPS1* and *GARNL3* in at least 156 unrelated patients with mild to severe idiopathic intellectual disability detected no causative mutations. Gene expression analyses in our patients demonstrated significantly reduced expression levels of *GARNL3*, *RALGPS1* and *STXBP1* only in patients with deletions of the corresponding genes. Thus, reduced expression of *STXBP1* was ruled out as a cause for seizures in our patient whose deletion did not encompass *STXBP1.*

**Conclusions:**

We suggest that microdeletions of this region on chromosome 9q cause a clinical spectrum including intellectual disability, developmental delay especially concerning speech, microcephaly, short stature, mild dysmorphisms, strabismus, and seizures of incomplete penetrance, and may constitute a new contiguous gene deletion syndrome which cannot completely be explained by deletion of *STXBP1*.

**Electronic supplementary material:**

The online version of this article (doi:10.1186/s13039-015-0178-8) contains supplementary material, which is available to authorized users.

## Background

Previously, microdeletions affecting the chromosomal region 9q33.3-q34.11 have been reported in association with early infantile epileptic encephalopathy 4 (EIEE4, or Ohtahara syndrome, OMIM #612164). The *STXBP1* gene was identified as a causative gene and loss-of-function mutations of *STXBP1* have been shown to cause EIEE4 [1], West syndrome [2], other seizure phenotypes [3], non-syndromic epilepsy [4], and intellectual disability (ID). *De novo* sequence mutations in *STXBP1* have also been reported to be associated with ID without epilepsy [5].

Here we present the clinical and genetic characterization of five patients whose common clinical features include intellectual disability (ID), psychomotor developmental delay (DD) with delayed or absent speech, muscular hypotonia, strabismus, dysmorphisms and other recurrent findings. For these patients, we have identified overlapping microdeletions of chromosome 9q33.3-q34.11 by molecular karyotyping. The smallest region of overlap (SRO) concerning these common clinical features contains only two RefSeq genes, which has implications regarding the potential role *STXBP1* may or may not play in our patients’ phenotypes.

## Results

### Case reports

#### Patient 1

Patient 1 is the first and only child of healthy non-consanguineous parents from Turkey with unremarkable family histories. She was born by vacuum extraction after 41 weeks of gestation with a birth weight of 3135 g (10–25^th^ centile, −1.1 S.D.), a length of 49 cm (3^rd^–10^th^ centile, −1.7 S.D.), and an OFC of 32 cm (<3^rd^ centile, −2.6 S.D.). Postnatal adaptation was normal (APGAR 10/10, umbilical cord pH 7.26). Exotropia of the left eye and bilateral pes supinatus were noted at the age of 1 month. Between 6 and 10 months of age, she displayed clear motor delays and muscular hypotonia. Cranial ultrasound examination at 8 months gave normal results, except for asymmetry of lateral ventricles. Hearing was normal.

**Fig. 1:**
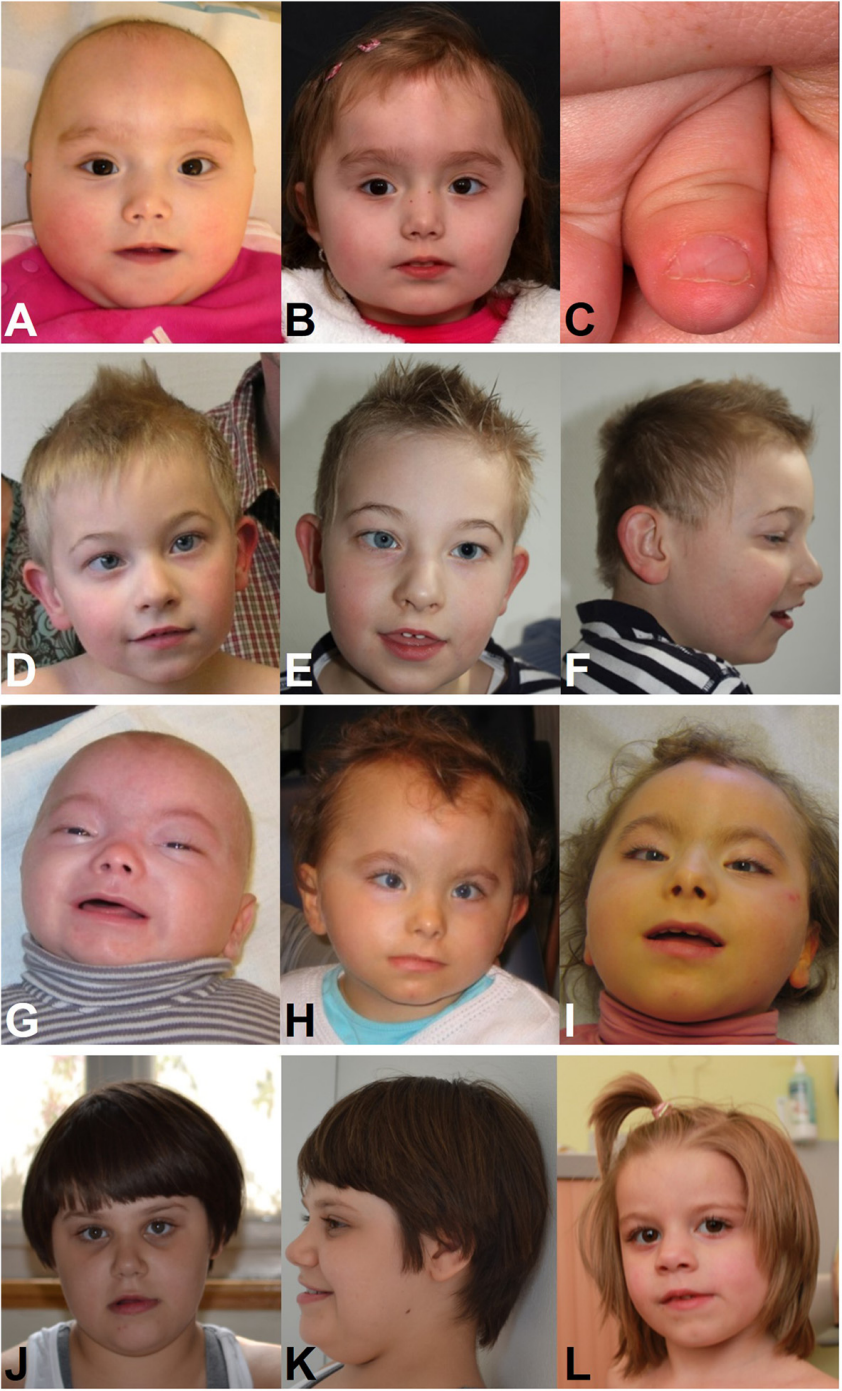
Facial phenotypes and nail abnormalities of patients with 9q33.3-q34.1 deletion. Facial phenotypes of patients 1–5 (**a**, **b** and **d**-**l**) and nail abnormalities of patient 1 (**c**). Patient 1 at the age of 11 months (**a**) and 35 months (**b**). Note round face, telecanthus, upslanting palpebral fissures, epicanthic folds, short nose, thin upper lip, low-set ears, tapering fingers, and ridged nail of the thumb (**c**). Patient 2 at the age of 5 years (**d**), and 6 years (**e**, **f**). Note prominent forehead, arched eyebrows, slightly upslanting palpebral fissures, and thin upper lip. Patient 3 at the age of 4 years (**l**). Note high frontal hairline, a short nose with anteverted nares and a dimpled chin. Patient 4 at the age of 5 months (**g**), 3 years (**h**), and 5 years (**i**). Note high frontal hairline, telecanthus, upslanting palpebral fissures, arched eyebrows, and thin upper lip. Patient 5 at the age of 11 years (**j**, **k**). Note round face, thin upper lip, and prominent lower lip

At the age of 10 months, her height and weight were normal [69 cm (3rd–10^th^ centile, −1.6 S.D.); 10.5 kg (90–97^th^ centile, +1.6 S.D.)], but she remained microcephalic [42.7 cm (<3^rd^ centile, −2.3 S.D.)]. Clinical genetic examination at 11 months revealed some craniofacial dysmorphism with cranial asymmetry (flat right occiput), round face, telecanthus, upslanting palpebral fissures, epicanthic folds, short nose, thin upper lip, low-set ears with attached earlobes, and a short neck. Her fingers were tapered with ridges of both thumb and index finger nails (Fig. 1a, c). Pes equinus was noted. She showed muscular hypotonia, especially of the trunk and shoulder girdle. She was able to bring her hands together as well as to her mouth and feet, as well as grasp toys and transfer them to the opposite side of her body. However, she was unable to roll over or support her upper body in the prone position. At the age of 20 months she weighed 14 kg (97^th^ centile, +2.0 S.D.), her height was 78.5 cm (3^rd^–10^th^ centile, −1.8 S.D.), and her OFC was 46 cm (10–25^th^ centile, −1.1 S.D.). In addition to the craniofacial dysmorphism described above she showed brachycephaly and a high frontal hairline. At the age of 20 months she was able to roll over but not to crawl. Language development was delayed with only few syllables. She showed strabismus and bruxism. Recurrent otitis was treated with transtympanic drains. The parents reported that EEG and ultrasonic examinations of the heart and kidneys were performed with normal results. Cerebral MRI scan at the age of 33 months showed a delay of myelination of about 3 months. At the age of 3 years, muscular hypotonia was very distinctive. The patient was unable to sit, stand, or walk. Her parents reported lack of pain sensation. She still displayed no seizures.

Conventional karyotyping and sequencing of the *RPS6KA3* (*RSK2*) gene yielded normal results.

#### Patient 2

Patient 2 is the first child of healthy and unrelated parents. He was delivered spontaneously after gestational week 40 + 4 with a birth weight of 2650 g (<3^rd^ centile, −2.3 S.D.), a length of 47 cm (<3^rd^ centile, −2.5 S.D.), and an OFC of 31.5 cm (<3^rd^ centile, −3.3 S.D.). Global developmental delay became apparent at 1 year. At 15 months he was able to turn from supine to prone. Clinical features including ataxia, repeatedly pathological EEG, and a cheerful manner suggested Angelman syndrome. No speech development was apparent. At the age of 5 years, he was able to crawl and to walk with assistance. His weight was 15 kg (<3^rd^ centile, −2.1 S.D.), his height 97 cm (<3^rd^ centile, −3.1 S.D.), and his head circumference 48 cm (<3^rd^ centile, −2.9 S.D.). He showed slight dysmorphisms such as a prominent forehead, arched eyebrows, slightly upslanting palpebral fissures, smooth philtrum, thin upper lip, and widely-spaced teeth, but no major dysmorphic stigmata (Fig. 1d).

Conventional karyotyping, subtelomere screening, methylation analysis at the 15q11.2 locus, and sequencing of *UBE3A* and *SLC9A6* gave normal results.

#### Patient 3

Patient 3 is a 6-year-old girl born as the first child of a French mother and an unknown father. Her 3-year-old maternal half-brother shows normal development. The mother, who was 15 years old at the birth of her daughter, has mild intellectual disability and poor language abilities. She has normal measurements without micro- or brachycephaly and no seizure history. Her only dysmorphisms are mildly anteverted nares and a dimpled chin.

Patient 3 was born at gestational week 35 with a weight of 2630 g (50^th^ centile), a length of 45 cm (25^th^ centile, −1.0 S.D.), and an OFC of 31 cm (10^th^ centile, −1.5 S.D.). Mild muscular hypotonia was noted as well as talus valgus. She started walking at the age of 14 months and walked without support at 22 months. Neither feeding difficulties nor obesity were reported in the first 2 years of life. There was no active speech development. Her recurrent otitis was treated repeatedly with transtympanic drains.

Upon clinical genetic examination at the age of 4 years and 5 months, her weight was 13 kg (3^rd^ centile, −2.0 S.D.), her height was 92 cm (3^rd^ centile, −2.0 S.D.), and her OFC was 45.5 cm (3^rd^ centile, −2.0 S.D.). She was microcephalic but not brachycephalic. A high frontal hairline, convergent strabismus, a short nose with anteverted nares and a dimpled chin were noted (Fig. 1l). Hands and fingers were normal. Language development was severely delayed with only few syllables and cries as vocalizations. She showed stereotypic hand movements and autistiform behavior. She suffered from seizures which were treated with Valproate. A cerebral MRI scan showed bilateral temporal-occipital pachygyria with heterogeneous “nodules” of grey cortex. Upon examination at the age of 6 years, her weight was 19.4 kg (25^th^ centile, −0.5 S.D.), her height was 105 cm (3^rd^ centile, −2.0 S.D.), and her OFC remained at 48 cm (3^rd^ centile, −2.0 S.D.). Severe intellectual disability, muscular hypotonia, and autistic behavior were still present. She showed bruxism and her language was limited to repeating single words. She had strabismus, a short nose with anteverted nares, and a flat face, as well as long and thin fingers and pes planus. At the age of 9 years, she was still reported to have no active speech and to have remained seizure-free under treatment for 20 months.

*FMR1* analyses by PCR and Southern blot and *MECP2* analysis (DHPLC) gave normal results.

#### Patient 4

Patient 4 is a 5 year and 6 months old girl born prematurely after 27 weeks of gestation because of a placental hematoma. Her unrelated parents are of French descent and there is no particular familial history of genetic disease. Birth weight was 950 g (65^th^ centile, +0.5 S.D., age-corrected), height was 34 cm (37^th^ centile, −0.5 S.D.), and OFC 24 cm (29^th^ centile, −0.5 S.D.). Bilateral talipes equinus and a patent ductus arteriosus which required surgical treatment were noted at birth. She had retinopathy of prematurity with vitreous hemorrhage as well as bilateral microphtalmia and alternate strabismus. At the age of 2 months, she presented with generalized seizures with persistent, almost daily crises which were resistant to treatment. A brain MRI gave normal results. Arm and forearm X-rays showed diffuse bone demineralization and radiocubital synostosis.

She was first referred for clinical genetic evaluation at the age of 5 months. Dysmorphic features including high frontal hairline, telecanthus, upslanting palpebral fissures, arched eyebrows, flat nasal bridge, thin upper lip, and retrognathism were noted (Fig. 1g). She also had bilateral 5th finger clinodactyly, absent thumb nails, and arched and abnormal nails of both index fingers. Her weight was 3625 g (5^th^ centile, −1.9 S.D., age-corrected), height 49.5 cm (1^st^ centile, −2.5 S.D., age-corrected), and OFC 35 cm (<3^rd^ centile, −3.2 S.D.). Standard karyotyping gave normal results. At last examination at the age of 5 years and 6 months (Fig. 1i), she still had developed no active language but showed eye contact and was able to smile. Sitting without support had not been acquired. Her weight was 16 kg (8^th^ centile, −1.8 S.D.), height 96 cm (below the 1^st^ centile, −3.5 S.D.), and her OFC 47 cm (2^nd^ centile, −2.2 S.D.). Nephropathy was diagnosed and CT scans at the age of 5 years and 6 months showed non-ossified and dislocated patellae, with bilateral patellar tendon hypoplasia.

#### Patient 5

Patient 5 was published previously without clinical detail [6]. She was the first child to non-consanguineous healthy Brazilian-Swiss parents after eight miscarriages and two unsuccessful IVF attempts. The family history was otherwise unremarkable. After an uneventful pregnancy, the girl was born at term by Cesarean section. At birth, her weight was 2500 g (3^rd^–10^th^ centile, −2.0 S.D.) and her length 48 cm (25–50^th^ centile, −1.2 S.D.); her head circumference was not documented.

As a neonate she presented with muscular hypotonia and subsequently with pronounced early developmental delay. She started sitting without support at 1 year of age and started walking at 4 years of age. She developed truncal and gait ataxia. Cerebral MRI and EEG exams at 6 years of age gave normal results. She never presented with clinical seizures. Sleeping issues were apparent, including trouble in falling asleep or to sleep through the night, and parasomnia with episodes of crying and screaming. Polysomnography at 10 years of age did not show EEG anomalies but confirmed episodes of arousal and pavor nocturnus. At the age of 11 years and 7 months, she spoke a few words. Auditory testing was normal. Except for strabismus, there were no visual impairments. Celiac disease was confirmed by biopsy and treated by diet. Toilet training has never been achieved.

At 11 years and 7 months of age, the patient presented with truncal obesity (OFC 53 cm, 25–50^th^ centile, −1.2 S.D.; length 137 cm, 10^th^ centile, −1.5 S.D.; weight 44 kg, 50–75^th^ centile, +0.6 S.D.). She was restless and hyperactive, limiting the overall physical exam. She did not have specific dysmorphic signs; a round face, round eyes with long eyelashes, strabismus, a short nose, a thin upper lip, and an everted lower lip were noted (Fig. 1j, k). Hands and feet were small with pes planus. Hypotonia and ataxia were evident.

Previous genetic testing for Angelman syndrome (methylation specific PCR) and karyotype analysis as well as standard metabolic testing results gave normal results.

### Chromosomal micro-array analyses

**Fig. 2:**
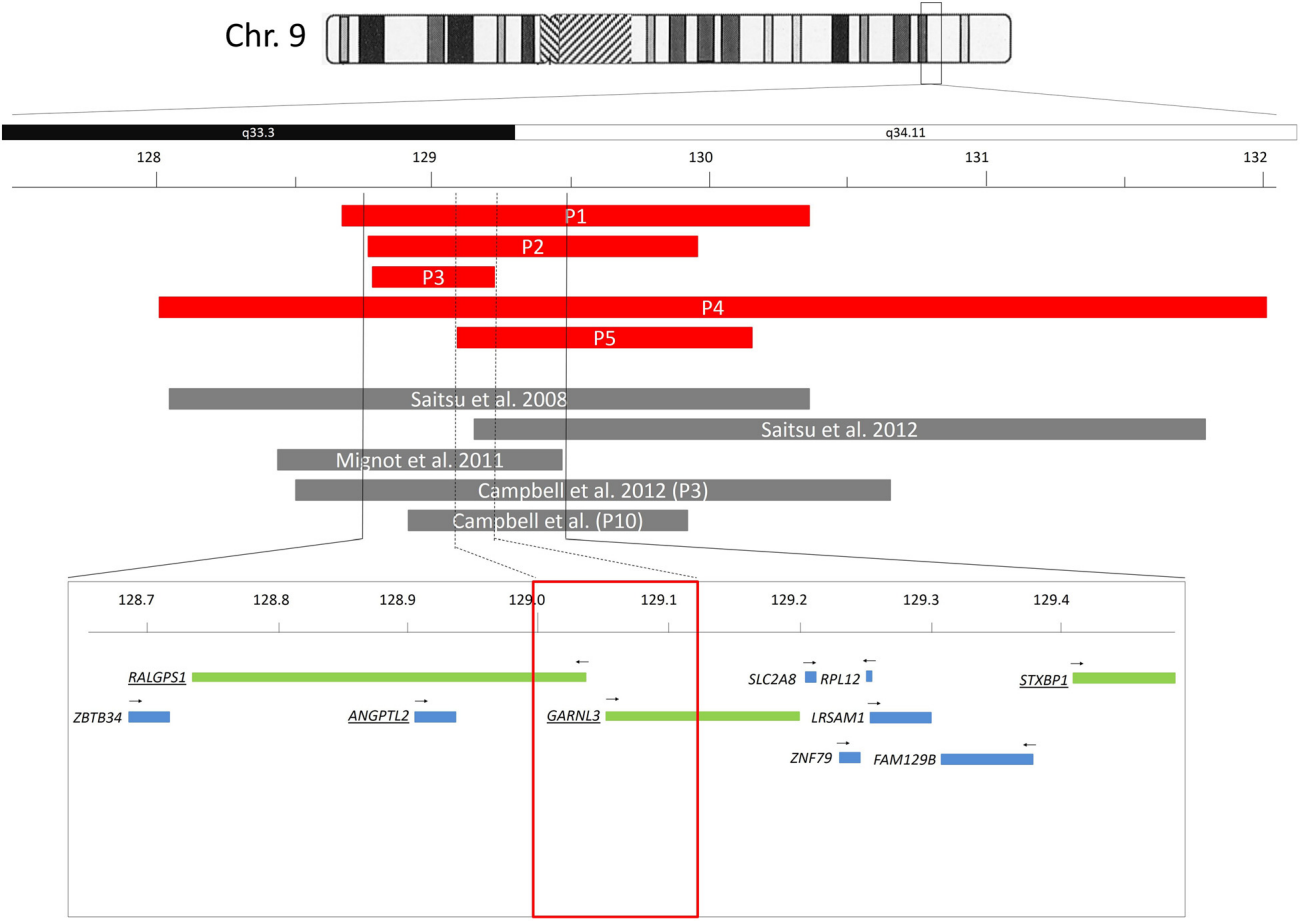
Schematic representation of the microdeletions. *Red bars*: Microdeletions of patients 1 through 5 are presented. *Grey bars*: previously published microdeletions. *Lower panel*: detail with smallest region of overlap (SRO, *dashed lines*, *red box*) and RefSeq genes with *black arrows* showing direction of transcription (*green*: genes analyzed by expression studies)

In patients 1, 2, 4 and 5, *de novo* deletions in 9q33.3-q34.11 ranging from 1.23 to 4.13 Mb were detected using various array platforms (Table 1, Fig. 2). Interestingly, the smallest deletion was detected in patient 3, whose 432-kb deletion was inherited from her mother who is reported to have mild ID. The smallest region of overlap (SRO) is defined by the distal boundary of the deletion in patient 3 and the proximal deletion boundary in patient 5, and includes only two RefSeq genes, *RALGPS1* and *GARNL3*. In patients 1, 2, 3 and 4, *ANGPTL2* is also deleted. Importantly, the SRO does not include the *STXBP1* gene in 9q34.11 (Fig. 2). No additional *de novo* CNVs or CNVs in known copy number-sensitive regions have been detected.

**Table 1:** Main clinical findings in patients with microdeletions of 9q33.3-q34.1

		Patient 1	Patient 2	Patient 3	Patient 4	Patient 5	Campbell et al. 2012 [11] (patient 3)	Campbell et al. 2012 [11] (patient 10)	Saitsu et al. 2008 [1] (patient 1)	Saitsu et al. 2012 [12] (Patient 2231^a^)	Mignot et al. 2011 [13] (patient 3)
Deletion size		1.76 Mb	1.20 Mb	432 kb	4.13 Mb	1.23 Mb	2.65 Mb	830 kb	2.10 Mb	2.85 Mb	3.3 Mb
ISCN 2013		arr [hg19] 9q33.3q34.11 (129,463,613–131,224,189) × 1	arr [hg19] 9q33.3q34.11 (129,630,976–130,833,333) × 1	arr [hg19] 9q33.3 (129,688,382–130,120,163) × 1	arr [hg19] 9q33.3q34.11 (128,870,221–132,995,660) × 1	arr [hg19] 9q33.3q34.11 (129,950,179–131,180,179) × 1	arr [hg19] 9q33.3q34.11 (129,473,714–131,633,299) × 1	arr [hg19] 9q33.3q34.11 (129,843,508–130,681,956) × 1	arr [hg19] 9q33.3q34.11 (129,060,488–131,199,630) × 1	arr [hg19] 9q33.3q34.11 (129,980,488–132,830,579) × 1	n.r. del 9q33.3–q34.11
Affected genes		*RALGPS1*, *ANGPTL2*, *GARNL3* + 48 RefSeq genes incl. *STXBP1*	*RALGPS1*, *ANGPTL2*, *GARNL3* + 28 RefSeq genes incl. *STXBP1*	*RALGPS1*, *ANGPTL2*, *GARNL3*	*RALGPS1*, *ANGPTL2*, *GARNL3* + 92 RefSeq genes incl. *STXBP1*	*RALGPS1*, *GARNL3* + 45 RefSeq genes incl. *STXBP1*	*RALGPS1*, *ANGPTL2*, *GARNL3* + 58 RefSeq genes incl. *STXBP1*	*RALGPS1*, *ANGPTL2*, *GARNL3* + 22 RefSeq genes incl. *STXBP1*	*RALGPS1*, *ANGPTL2, GARNL3* + 50 RefSeq genes incl. *STXBP1*	*RALGPS1*, *GARNL3* + 86 RefSeq genes incl. *STXBP1*	*RALGPS1*, *ANGPTL2*, *GARNL3* + > 9 RefSeq genes incl. *STXBP1*
Origin		*De novo*	*De novo*	Maternal	*De novo*	*De novo*	*De novo*	*De novo*	*De novo*	*De novo*	*De novo*
Age		3 y	5 y	6 y	5 y, 6 mo	11 y, 7 mo	6 y	6 y	2 y, 11 mo	1 y, 7 mo	10 y
Sex		Female	Male	Female	Female	Female	male	Male	Female	Male	Female
Birth parameters	Length	<10^th^ centile	<3^rd^ centile	25^th^ centile	10^th^ centile	ND	n.r.	n.r.	−2.3 SD	Normal	~10^th^ centile (est.)
	Weight	10^th^ centile	3^rd^ centile	50^th^ centile	10^th^ centile	0.4^th^–2^nd^ centile	n.r.	n.r.	−2.4 SD	Normal	~10^th^ centile (est.)
	OFC	<3^rd^ centile	<3^rd^ centile	10^th^ centile	10^th^ centile	ND	n.r.	n.r.	−0.8 SD	Normal	ND
Growth parameters (at examination)	Height	10^th^ centile	4.5 cm < 3^rd^ centile	~6 cm < 3^rd^ centile	3^rd^ centile	0.4^th^ centile	n.r.	n.r.	<−2SD^a^	n.r.	10^th^ centile
	Weight	25–50^th^ centile	10^th^ centile	10–25^th^ centile	10^th^ centile	25^th^ centile	n.r.	n.r.	<−2SD^a^	n.r.	75–90^th^ centile
	OFC	~1.5 cm < 3^rd^ centile	2 cm < 3^rd^ centile	~0.5 cm < 3^rd^ centile	10^th^ centile	<0.4^th^ centile	n.r.	n.r.	40.0 cm (<< 3^rd^ centile)^a^	−3SD	50–75^th^ centile
Microcephaly		+	+	+	–	+	+	n.r..	n.r.	+	+
Intellectual disability		+	+	+	+	+	+	+	+	+	+
Speech		Delayed (only syllables)	No speech	No speech (some syllables and cries)	No speech	Delayed (few words)	Severely impaired	Severely impaired	No speech	n.r.	Delayed (few words)
Walking		No (no crawling/sitting/standing)	Yes (only with assistance)	Yes (walking age 22 mo)	No	Yes (walking age 4 y)	n.r.	n.r.	No	n.r.	No
Seizures		–	–	+	+	–	+	–	+	+	+
EEG		Pathologic	Pathologic	Normal	Pathologic	Normal	Right temporal spikes	Normal	Suppression-burst pattern	Suppression-burst pattern	Disorganized background activity, multifocal spikes, spike and spike-wave bursts
Brain MRI		Mildly delayed myelination	Delayed myelination, unilateral temporal closed lip schizence-phaly	Bilateral temporo-occipital pachygyria with heterogenous “nodules” of grey	Normal	Normal	Chiari type I malformation	Normal	Cortical atrophy, diffuse hypomyeli-nation, thin corpus callosum, cerebellum and brain stem atrophy (at 12 mo)	Thin corpus callosum, relatively small cerebellum (2 mo)	Global atrophy of cerebral hemispheres
Muscular hypotonia		+	+	+	+	+	+	+	+	n.r.	n.r.
Dysmorphisms		*Round face*, *high frontal hairline*, telecanthus, *upslanting palpebral fissures*, *short nose* with depressed bridge, *thin upper lip*, low-set ears with attached lobules, short neck, *tapering fingers with ridged nails*, *talipe equinus*	*Arched eyebrows*, *slightly upslanting palpebral fissures*, smooth philtrum, widely-spaced teeth, *thin upper lip*	*Short nose*, anteverted nares, *high frontal hairline*, dimple chin, talus valgus bilateral	*Arched eyebrows*, telecanthus, horizontal palpebral fissures, flat nasal bridge, retrognathia, bilateral 5th finger clinodactyly, 1st fingers: absent nails, *talipe equinus*	*Round face*, round eyes, long eyelashes, *short nose*, *thin upper lip*, slightly everted lower lip, small hands and feet	Dysmorphic features	Mild dysmorphic features	n.r.	Midface hypoplasia^a^	dysmorphic features
Ocular findings		*Strabismus*	*Strabismus*, hypermetropia	*Strabismus*	*Strabismus*, retinopathy of prematurity, bilateral microphtalmia	*Strabismus*	*Strabismus*	–	n.r.	n.r.	n.r.
Behavior		Bruxism	Cheerful mannerism	Autistic features, stereotypic hand movements	ND	Hyperactive, sleep difficulties	n.r.	n.r.	n.r.	n.r.	Stereotypies
Other findings		Recurrent otitis media, lack of pain sensation	Hypotonic-ataxic movement disorder	Recurrent otitis media, failure to thrive	Forearm malformation, non-ossified and dislocated patellae, nephropathy	Trunk and gait ataxia, pes planovalgus, celiac disease, overweight		Ataxia	Spastic quadriplegia	Cleft lip/palate, ventricular septal defect, overlapping fingers, small penis, spastic quadriplegia, multiple arthrogryposis	Absent thumbnails, nails of 2nd fingers hypoplastic

Summary of patient phenotypes and deletions

*est.* estimated, *ND* not determined, *n.r.* not reported, *OFC* occipitofrontal circumference, *SD* standard deviation, + present, – absent, *y* years, *mo* months

^a^Personal communication. Recurrent dysmorphisms and ocular findings are italicized

### Sanger sequencing of SRO genes *RALGPS1* and *GARNL3*

Every exon including exon/intron boundaries of all known protein-coding RefSeq isoforms of *GARNL3* and *RALGPS1* has been studied in at least 156 of the 192 ID/DD patients. The sequencing revealed eight rare variants in *GARNL3* and none in *RALGPS1* (Additional file 1: Table S1). All *GARNL3* variants were present in a heterozygous state in either one or two patients with ID/DD (minor allele frequencies/MAF < 0.58 %). Four variants caused amino acid changes whereas the remaining four variants were synonymous. In total, four of the eight *GARNL3* variants were listed in dbSNP build 134 and were also present in the NHLBI GO-ESP cohort (Exome Variant Server, NHLBI GO Exome Sequencing Project (ESP), http://evs.gs.washington.edu/EVS/; July 2015). The remaining four variants were followed up by pedigree analyses. All variants had been inherited from an unaffected parent (Additional file 1: Table S1), suggesting no obvious association between the variants and the occurrence of ID.

### Expression analyses of SRO genes and *STXBP1*

We performed gene expression studies with predesigned Taqman gene expression assays for *GARNL3*, *RALGPS1*, *STXBP1*, and *ANGPTL2* on whole blood RNA of five patients with microdeletion and of five unaffected control persons. The expression level of *ANGPTL2* which is not contained in the SRO but in four of the detected microdeletions including the smallest one was determined to be too low in whole blood RNA to obtain results.

**Fig. 3:**
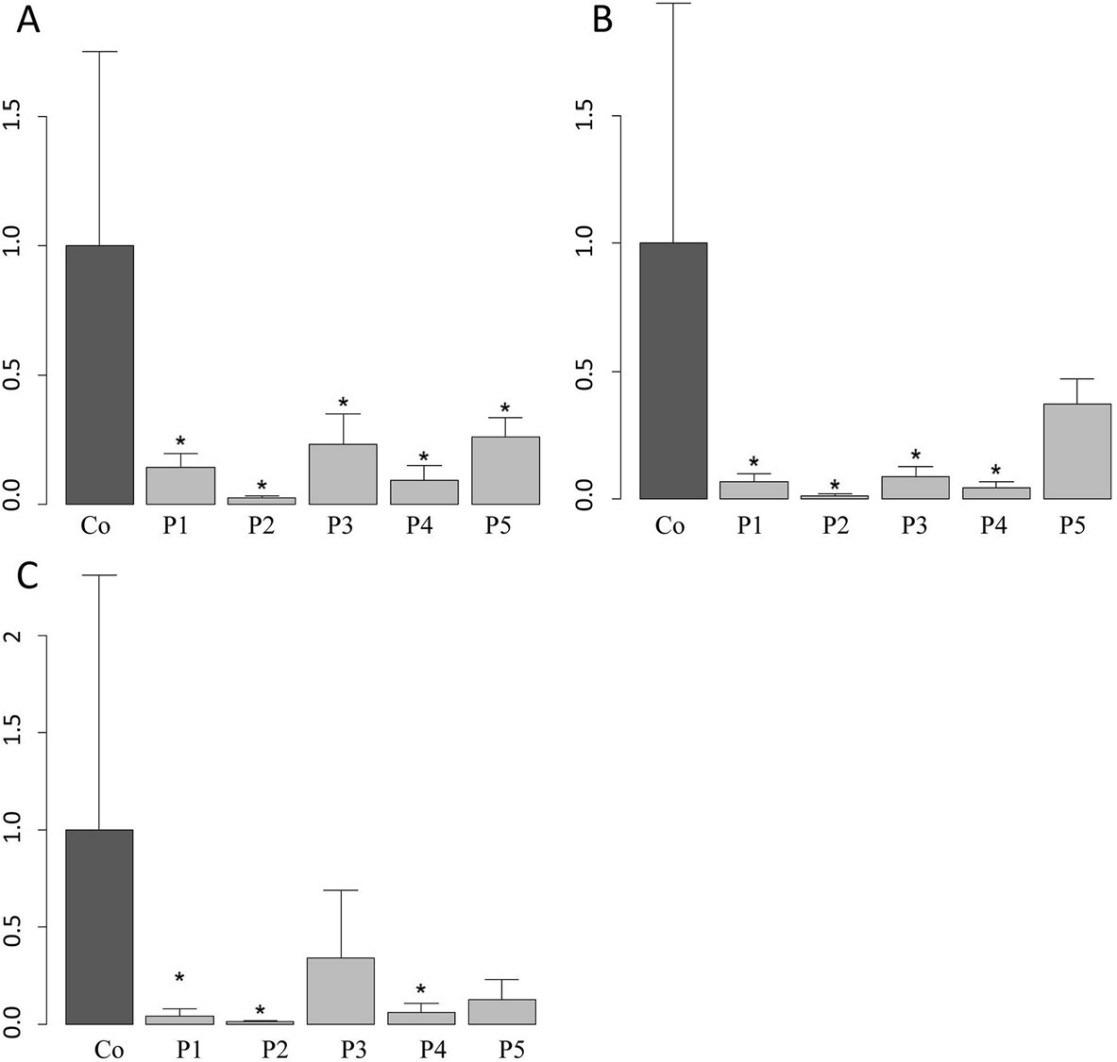
Results of expression analyses for **a** *GARNL3*, **b** *RALGPS1*, and **c** *STXBP1*. The mean expression of five unaffected controls is set to 1.0 (*dark grey bar*). The expression level of the patients (*light grey*) is set in relation to the mean of the five controls. *Error bars* are based on the data obtained over three experiments. *Asterisks* mark significant expression differences between patients and controls (*p* < 0.05, Wilcoxon-test)

Compared to the mean of five unaffected controls, the expression levels of *GARNL3* were significantly (*p* < 0.05) reduced in all five patients (Fig. 3a). For *RALGPS1*, the expression was reduced significantly in patients 1 through 4, but not in patient 5 (Fig. 3b). *STXBP1* showed a significantly reduced expression in patients 1, 2, and 4, while its expression in patients 3 and 5 was not reduced significantly (Fig. 3c).

## Discussion

Array CGH is now widely used in cytogenetics centers for postnatal constitutional genome analysis and in research for the identification of new causative microdeletion and microduplication syndromes or copy number variations (CNVs) and has alos been instrumental in the identification of new candidate genes for e.g. intellectual disability (ID) [7–9].

Several microdeletions involving the chromosomal region 9q33.3-q34.11 have been reported and seem to be associated with a range of clinical phenotypes including, but not limited to, ID, developmental delay (DD), and seizures [10, 11]. Several studies identifying such microdeletions had in fact been focused on patients with diverse seizure phenotypes [1, 12] or aimed at identifying mutations including CNVs involving *STXBP1. De novo* missense, nonsense, frameshift, and splice-site mutations, as well as genomic deletions of *STXBP1*, have been found in association with Early Infantile Epileptic Encephalopathy 4 (EIEE4; OMIM #612164) and other epilepsy types [13], such that *STXBP1* haploinsufficiency is the accepted cause of these epileptic phenotypes. Consequently, all the published microdeletions of 9q33.3-q34.11 contained *STXBP1* and all published microdeletion carriers suffered from seizures.

Here, we report five novel patients with microdeletions of 9q33.3 only or 9q33.3-q34.11 which vary greatly in size (0.4–4.1 Mb; Table 1) and gene content (3–95 RefSeq genes). The smallest deletion reported here encompasses only three RefSeq genes (*RALGPS1*, *ANGPTL2*, and *GARNL3*). In spite of the differences between the breakpoints of their deletions, the patients show considerable clinical similarities. All five patients have ID and DD with pronounced speech delay or impairment, muscular hypotonia, and strabismus. Microcephaly and postnatal (borderline) short stature are present in four of the patients, while only two of them display seizure phenotypes. Other clinical findings shared by at least two patients include ataxia phenotypes (patients 2 and 5), recurrent otitis media (patients 1 and 3), and unspecific brain MRI findings (patients 1, 2, and 3) including delayed myelination in two patients. Common dysmorphisms include a round face (patients 1 and 5), upslanting palpebral fissures (patients 1 and 2), a short nose (patients 1, 2, and 5), and a thin upper lip (patients 1, 2, and 5).

In addition, two of the patients reported here also display typical findings of Nail-Patella syndrome (NPS, OMIM # 161200), including talipe equinus, reduced pain sensation, high frontal hairline, and ridged nails in patient 1 (Fig. 1c) and bilateral 5th finger clinodactyly, missing nails of the first fingers, talipe equinus, non-ossified and dislocated patellae, and typical forearm malformations in patient 3. However, the gene known to be responsible for NPS, *LMX1B*, only appears to be deleted in patient 4, while the minimal deletion interval in patient 1 starts 302 base pairs downstream from *LMX1B*. However, as the deletion breakpoints have not been sequenced, it is possible that the deletion in patient 1 in fact could extend into the coding region of *LMX1B*, causing features of NPS.

A comparison of the clinical picture of the patients with microdeletions reported here with published carriers of *STXBP1* loss-of-function point mutations or intragenic deletions yields several similarities. ID and DD with language delay, muscular hypotonia, seizures, ataxia, and brain MRI findings including hypomyelination were also present in patients with *STXBP1* mutations [3–5, 14]. The reduced penetrance regarding seizures reported by Hamdan and colleagues is confirmed and even emphasized by our findings, since only one out of four carriers with *STXBP1*-encompassing microdeletions reported here suffered from seizures. In contrast to these commonalities, several clinical findings are exclusively present in our patients with microdeletions. To our knowledge, microcephaly, short stature, and strabismus have not been reported in carriers of *STXBP1* point mutations or of deletions affecting *STXBP1* only. This may also be true for at least some of our patients’ dysmorphisms, but in most studies of *STXBP1* point mutations, the presence or absence of dysmorphisms is not mentioned. Only Hamdan and colleagues explicitly state that there were no dysmorphic features present in their three patients with truncating *STXBP1* mutations. Thus, it may be assumed that the common findings–microcephaly, short stature, and possibly strabismus–which are exclusive to the five patients with microdeletions reported here are unrelated to *STXBP1* and associated instead with the smallest region of overlap (SRO) of their deletions. Importantly, our patient 3 presents with seizures although her deletion does not include *STXBP1* and her *STXBP1* expression is not reduced. We thus hypothesize that her seizures are unrelated to *STXBP1* and may instead be caused by the deletion of the SRO.

We are aware of five previously published patients with microdeletions including these SRO genes (Table 1). For three of these patients, seizures were the primary indication for referral [1, 12, 13], while two of the patients were referred for array CGH for a diverse set of clinical indications [11]. All patients with corresponding clinical data displayed ID and DD with severe language delay and muscular hypotonia. Eight out of nine patients had postnatal microcephaly and five out of seven postnatal short stature or borderline short stature. Seven out of ten patients had brain MRI abnormalities and six out of ten suffered from seizures. When excluding the three patients investigated primarily because of their seizures, to minimize any ascertainment bias, only three out of seven patients suffered from seizures. Dysmorphisms are present in most patients and some dysmorphic features were seen in up to three of the patients presented here. However, there does not seem to be a readily recognizable dysmorphism pattern.

The SRO is localized in distal 9q33.3 and only includes two RefSeq genes, *RALGPS1* and *GARNL3.* In patients 1 through 4, the *ANGPTL2* gene is also deleted (Fig. 2). The clinical and genomic evidence may point to the existence of a 9q33.3 microdeletion syndrome which may be unrelated to *STXBP1* haploinsufficiency. Until now, none of the three genes has been associated with disease. *RALGPS1* (Ral GEF with PH domain and SH3 binding motif 1), formerly known as *RalGEF2*, encodes a guanine nucleotide exchange factor or GEF for Ral (de Bruyn et al. 2000 [15]). Ral (RalA and RalB) is a small GTPase of the Ras family implicated in the control of cell proliferation, differentiation, cytoskeletal organization, and vesicular transport. One of the mechanisms of Ral activation is the direct binding of active Ras to Ral-specific GEFs such as RALGPS1 [15]. *RALGPS1* expression is rather ubiquitous with the highest expression levels in brain, heart, kidney, adrenal gland, and colon. The evidence for *RALGPS1* haploinsufficiency is equivocal: the Residual Variation Intolerance (RVI) score, which quantifies gene intolerance to functional mutations [16] is intermediate (−0.293801652, corresponding to the 32.9th percentile). As a comparison, the average RVI score for known developmental disorders is lower (0.56; 19.54th percentile). On the other hand, its low haploinsufficiency score (HI) of 8.9 % indicates a high probability for haploinsufficiency being pathogenic [17].

Very little is known about the *GARNL3* (Homo sapiens GTPase activating Rap/RanGAP domain-like 3) gene. It appears to be involved in the regulation of small GTPase-mediated signal transduction and human *GARNL3* is rather ubiquitously expressed with the highest expression in brain [18]. Rap/ran-GAP domains are found in GTPase activating proteins (GAP) responsible for the activation of nuclear Ras-related regulatory proteins Rap1, Rsr1 and Ran. Again, the evidence for *GARNL3* haploinsufficiency is equivocal with a very low RVI score of −1.082044518 (7.2th percentile) and a rather high haploinsufficiency score (HI) of 33.6 %.

ANGPTL2 (Homo sapiens angiopoietin-like 2, also known as ARP2 for angiopoietin-related protein-2) is discussed here although it is not contained in the SRO because it is deleted in four out of five microdeletions presented here, including the smallest one. It is a secreted glycoprotein that has been implicated in angiogenesis, inflammation, and atherosclerosis, as well as enhancing the survival of human hematopoietic stem cells [19]. It is a member of the Angiopoietin vascular endothelial growth factor family and is largely specific for vascular endothelium. Angptl2 is expressed in the heart, adipose tissue, stomach, small intestine, colon, ovary, uterus, spleen, striated muscle, and, at lower levels, in other tissues and Angptl2 is secreted by different cell types such as adipocytes, endothelial cells, macrophages, keratinocytes and cancer cells [19, 20]. In zebrafish embryos, Zangptl2 expression was detected in the yolk sac extension and in posterior spinal cord during early development and shown to diminish gradually as development proceeded [21]. During chick embryogenesis, Angptl2 was detected at E3 in the hindbrain and at E4 in neuroepithelium of the forebrain and hindbrain and also in the heart [22]. As part of the Arp2/3 complex, ANGPTL2 interacts directly with the WAVE (Wiskott–Aldrich syndrome protein family verprolin-homologous protein) complex to induce actin cytoskeleton changes and is involved in the regulation of axon growth [23]. Regarding haploinsufficiency prediction, both the RVI score of −0.867014353 (10.7th percentile) and a low HI score of 6.9 % point to *ANGPTL2* haploinsufficiency being pathogenic.

In contrast to contiguous gene syndromes such as Williams syndrome, several other microdeletion syndromes have been shown recently to be caused mainly by haploinsufficiency of a single responsible gene such as *MEF2C* in 5q14.3 microdeletion syndrome [24] or *SHANK3* in Phelan-McDermid syndrome [25]. We were interested to determine which scenario applies to microdeletion 9q33.3 syndrome. Overall, neither expression patterns nor functional data nor the RVI and HI scores pointed to one of the SRO genes as the main or even single responsible gene for the 9q33.3-q34.1 microdeletions reported here. Consequently, we analyzed the expression levels of *RALGPS1, GARNL3*, and *ANGPTL2* to gain possible evidence for their haploinsufficiency. We included *STXBP1* in our expression studies in order to investigate the possibility of a reduced expression caused by long-range position effects of the deletion in patient 3 which did not include *STXBP1.* The gene expression studies for *GARNL3*, *RALGPS1*, *ANGPTL2*, and *STXBP1* on whole blood RNA of patients 1 through 5 demonstrated significantly reduced expression levels of the SRO gene *GARNL3* in all patients. The expression level of the non-SRO gene *ANGPTL2* in whole blood RNA was too low to be analyzed. *RALGPS1* expression was reduced significantly in patients 1 through 4 but not in patient 5 whose deletion did not encompass the gene. *STXBP1* did not show a significantly reduced expression in patient 3, in whom *STXBP1* was not deleted, thus excluding a possible long-range position effect for her deletion. Our expression studies did not narrow down the causative genes by providing evidence for haploinsufficiency of only one of the SRO genes. Thus, we performed Sanger sequencing of the SRO genes *RALGPS1* and *GARNL3* in more than 156 patients with ID and DD in order to find possible evidence of loss-of-function mutations in one of these genes as a frequent cause for ID and DD. However, all detected variants were either known single nucleotide polymorphisms listed in dbSNP or had been inherited from an unaffected parent. Thus, no causative mutations were detected and deleterious sequence mutations in *RALGPS1* or *GARNL3* are apparently not a common cause of ID and DD.

## Conclusions

In summary, we present evidence for a novel 9q33.3 microdeletion syndrome consisting of ID, DD with pronounced speech delay, muscular hypotonia, strabismus, and incompletely penetrant microcephaly, short stature, and seizures. The smallest region of deletion overlap affects only two coding genes, *RALGPS1 and GARNL3* and is localized in close proximity to the known ID / epilepsy gene *STXBP1.* The seizure, ID, and DD phenotype in the deletions described here is at least partly independent from *STXBP1*, as shown by microdeletion sizes and expression studies. Follow-up studies on additional patients are needed to determine if this syndrome is caused by the deletion of only one of the candidate genes *RALGPS1* and *GARNL3*, or if it constitutes a contiguous gene syndrome.

## Methods

### Clinical reports

Investigations were performed in accordance with the protocols of the Declaration of Helsinki and were approved by the Ethikkommission der Medizinischen Fakultät der Universität Bonn (lfd. Nr. 120/99, 131/08). Consent to participate was obtained from the parents for all participants. The retrospective reports of the microdeletions which were detected during routine array diagnostics did not require ethics committee approval at the respective institutions. Written consent for the publication of clinical photographs was obtained from the parents for all five patients.

### Array studies and verification

For patient 1, peripheral blood genomic DNA was analyzed using an Illumina HumanOmni1-Quad array according to the manufacturer’s instructions (Illumina, Inc., San Diego, CA, USA). CNV calling was performed as published previously [26, 27]. Real-time quantitative PCR with three independent primer pairs was performed to confirm the deletion in the patient and to test for the presence of the deletion in parental DNA as published previously [26, 27]. Primer sequences are available upon request.

For patient 2 and his parents, peripheral blood genomic DNA was analyzed according to manufacturer’s instructions with Affymetrix Genome-Wide Human SNP Arrays 6.0 (Affymetrix, Santa Clara, CA, USA) with an average distance of 1.3 kb between neighboring probes. Genotypes were called with Affymetrix Genotyping Console Software v3 (GTC) using the Birdseed algorithm with the default calling threshold of 0.5 and a prior size of 10,000 bases in a simultaneous analysis of the patient-parents trio. Interpretation was originally based on Human Genome Build 36 (NCBI).

Peripheral blood genomic DNA from patients 3 and 4 was analyzed using Agilent Human Genome CGH 44B microarrays (Agilent, Santa Clara, CA, USA) according to the manufacturer’s protocols. Female genomic DNA (Promega, Madison, WI, USA) was used as reference and quantitative PCR for verification and segregation analyses was performed with QuantiTect SYBR Green PCR Kits (Qiagen, Valencia, CA, USA).

Microarray analysis of genomic DNA from patient 5 was performed using the 720 K whole genome tiling NimbleGen CGH array (Nimble-Gen®; Roche NimbleGen Inc, Madison, Wisconsin, USA). Tiling array version Human CGH 3 × 720 K WG-T v3.0 array which contains 720,000 probes with a median probe spacing of 2509 bp was applied. The effective average resolution is approximately 50 kb for a required minimum of 10 consecutive aberrant oligonucleotides. Labeling and hybridization of test and reference DNA was performed according to the manufacturer’s protocols. Two-color scanning was performed with an MS 200 Scanner (NimbleGen®; Roche NimbleGen Inc, Madison, Wisconsin, USA). Microarray images were acquired using the MS 200 software. Data extraction, analysis, and visualization were performed using the NimbleScan™ version 2.5 and SignalMap™ version 1.9 software. Verification in the proband and segregation analyses in the parents were performed by fluorescence-in-situ hybridization (FISH) using the locus-specific BAC clone RP11-356B19 (9q34.11; BlueGnome, Cambridge, UK).

### Sanger sequencing of SRO genes *RALGPS1 *and *GARNL3*

Mutational screening of *RALGPS1* and *GARNL3* by Sanger sequencing was performed on 192 individuals seen at the University Medical Genetics Clinics in Bonn, 96 of whom had mild ID/DD and 96 of whom had moderate to severe ID/DD. This study group consisted of 66 females (34 %) and 126 males (66 %). A total of 82 individuals showed no or only negligible dysmorphisms (43 %), while 109 had dysmorphisms (57 %); of the latter group, 40 patients also showed organ malformations (21 %). Standard evaluation consisted of detailed clinical investigation, conventional karyotyping, and the exclusion of clinically recognizable syndromes with known etiology. The presence of Fragile X syndrome had been excluded in almost all patients. Previous molecular karyotyping with Illumina microarrays (550, 610, 660 K or Omni1-Quad) had identified no pathogenic aberrations. The study was approved by the Institutional Review Board of the University Hospital of Bonn, and informed consent was obtained for all participants.

To target the coding regions of *RALGPS1* and *GARNL3* and flanking intronic sequence (at least 15 bp), primers were designed using the online program Primer3 (http://primer3.ut.ee/). All known protein-coding RefSeq isoforms were analyzed: NM_014636, NM_001190728, NM_001190729 and NM_001190730 for *RALGPS1*, NM_032293 and NM_001286779 for *GARNL3*. Sequencing was performed with either genomic or whole genome amplified DNA (REPLI-g WGA kit, Qiagen, Hilden, Germany) of the 192 patients with ID/DD using standard procedures. Amplicons were sequenced bi-directionally using the BigDye Terminator v3.1 Cycle Sequencing Kit (Applied Biosystems, Foster City, California, USA). The fluorescently labeled fragments were analyzed on a capillary sequencing system (3130XL Genetic Analyzer, Applied Biosystems). Sequences were analyzed using SeqMan II software (DNAStar, Madison, WI, USA). All primer sequences as well as PCR conditions are available upon request. The frequency in control cohorts was analyzed for all detected variants using dbSNP build 142 and the NHLBI GO-ESP cohort (Exome Variant Server, NHLBI GO Exome Sequencing Project (ESP), http://evs.gs.washington.edu/EVS/; July 2015). The *in silico* tool CADD (Combined Annotation Dependent Depletion, http://cadd.gs.washington.edu/, [28]) was employed to predict pathogenicity.

### Expression analyses of *RALGPS1*, *GARNL3*, *ANGPTL2*, and *STXBP1*

Expression analyses for *RALGPS1, GARNL3*, *ANGPTL2*, and *STXBP1* were performed for microdeletion patients 1 through 5 and five unaffected control persons. RNA of the five patients and the five unaffected controls was extracted from whole blood using the PAXgene Blood system (Qiagen, Hilden, Germany). RNA was reverse transcribed using Superscript II (Invitrogen, Carlsbad, CA, USA). To measure expression levels, predesigned TaqMan® gene expression assays (Applied Biosystems, Foster City, CA, USA; Hs01115436_m1; Hs00171912_m1; Hs01060483_m1; Hs01119036_m1) were used. The gene expression analysis was performed in triplicate, with each reaction containing 2 μl of cDNA template in a 10 μl reaction volume, on a LightCycler® 480 Real-Time PCR System (Roche, Mannheim, Germany). The cycling conditions were as follows: 50 °C for 2 min, denaturation at 95 °C for 10 min followed by 40 cycles at 95 °C for 15 s, and a combined annealing and extension step at 60 °C for 60 s. Duplex reactions contained both target (FAM) and endogenous control (VIC) probes and primers. Two different endogenous controls (Human B2M (beta-2-microglobulin) Endogenous Control; Human PPIA (cyclophilin A) Endogenous Control; TaqMan® Endogenous Controls, Applied Biosystems, Foster City, CA, USA) were assayed in parallel and in separate duplex reactions. Normalized results were compared to the results of five unaffected controls. Each triplicate experiment was performed twice. P values were calculated using a two-sided Wilcoxon test.

## Additional file

Additional file 1: Table S1. Sequencing results of *GARNL3* in 192 patients with ID/DD.
