# Supplementary material for: Microdeletions in 9q33.3-q34.11 in five patients with intellectual disability, microcephaly, and seizures of incomplete penetrance: is STXBP1 not the only causative gene?
Source: Mol Cytogenet. 2015 Sep 29;8:72. doi: 10.1186/s13039-015-0178-8 (PMC4587785; doi:10.1186/s13039-015-0178-8)
Supplement: Additional file 1: Table S1. — Sequencing results of GARNL3 in 192 patients with ID/DD. [file 13039_2015_178_MOESM1_ESM.docx]

**Supplementary Table 1: Sequencing results of *GARNL3* in 192 patients with ID/DD.** In total, we identified eight variants in *GARNL3*. Four of these are listed in dbSNP build 142 (column “rsID”) and have been observed in NHLBI GO-ESP (column “MAF ESP EA %”). The remaining four variants were analyzed for a parental origin and were also present in an unaffected parent (column “Parental Origin”).

| **Gene** | **Chr. Position^1^** | **rsID** | **Allele^2^** | **Protein Change^3^** | **MAF ID/DD cohort % (N)**^4^ | **MAF ESP EA %**^4^ | **Parental Origin** |
| --- | --- | --- | --- | --- | --- | --- | --- |
| *GARNL3* | chr9:130,095,372 | rs141092281 | G/A | synonymous | 0.27 (1) | 0.01 | NA |
| *GARNL3* | chr9:130,104,605 | rs142937906 | T/A | p.L415M / p.L393M | 0.30 (1) | 0.07 | NA |
| *GARNL3* | chr9:130,116,658 | rs75324173 | C/G | p.D576E / p.D554E | 0.28 (1) | 1.40 | NA |
| *GARNL3* | chr9:130,119,547 | - | C/T | p.P662L / p.P640L | 0.30 (1) | - | maternal |
| *GARNL3* | chr9:130,145,742 | - | C/T | synonymous | 0.27 (1) | - | maternal |
| *GARNL3* | chr9:130,147,319 | rs369247689 | G/A | synonymous | 0.58 (2) | 0.01 | NA |
| *GARNL3* | chr9:130,147,348 | - | T/C | p.M771T / p.M749T | 0.29 (1) | - | paternal |
| *GARNL3* | chr9:130,151,331 | - | G/A | synonymous | 0.28 (1) | - | paternal |

^1^ Chromosomal (Chr.) position according to genome build GRCh37 / hg19.

^2^ Forward strand alleles with the major allele listed first

^3^ Protein changes are given for both affected transcripts NM_032293 / NM_001286779

^4^ Minor allele frequency (MAF) is shown for the studied cohort (ID/DD cohort) and for the European American (EA) NHLBI GO-ESP cohort (ESP EA). For the ID/DD cohort the number of patients carrying the variant in the heterozygous state is given in brackets (N)

^5^ NA, not analyzed
